# Early Detection of Covid-19 in Canadian Provinces and its Anticipatory Measures for a Medical Emergency

**DOI:** 10.1007/s42979-020-00347-0

**Published:** 2020-10-19

**Authors:** Vikram Kumar Kamboj, Chaman Verma, Anish Gupta

**Affiliations:** 1 Department of Electrical and Computer Engineering, Schulich School of Engineering, University of Calgary, Calgary, Canada; 2 Department of Media and Educational Informatics, Eötvös Loránd University, Budapest, Hungary; 3 Domain of Power System, School of Electronics and Electrical Engineering, Lovely Professional University, Phagwara, India; 4 ABES Engineering College, Ghaziabad, India

**Keywords:** ARIMA, Canadian province, Covid-19, Decease, Confirmed

## Abstract

The spread of COVID-19 is incearsing day by day and it has put the entire world and the whole humankind at the stack. The assets of probably the biggest economies are worried because of the enormous infectivity, and transmissibility of this ailment. Because of the developing extent of the number of cases and its ensuing weight on the organization and wellbeing experts, some expectation strategies would be required to anticipate the quantity of evidence in the future. In this paper, we have utilized time series forecasting approach entitled autoregressive integrated moving average, and bend fitting for the forecast of the quantity of COVID-19 cases in Canadian Province for 30 days ahead. The estimates of different parameters (number of positive cases, number of recouped cases, and decrease cases) got by the proposed strategy is exact inside a specific range, and will be a beneficial apparatus for overseers, and wellbeing officials to organize the clinical office in the distinctive Canadian Province.

## Introduction

In the unexpected pandemic (Covid-19), every human being is living in a fearful environment, and obviously, it is spreading out rapidly and effected the human’s life. In December 2019. All world is confronting a century pandemic Coronavirus, which has been spreading at an extremely high fast speed from the finish of 2019. Presenting in December 2019, right off the bat, it had tainted a Chinese human in Wuhan on 30th January 2020, China [1]. The official declaration of this novel pneumonia episode a “worldwide pandemic” on the eleventh walk by the World Health Organization (WHO) (Wang et al. [1]) and given another name Covid-19. Covid-19 has been moved from creature to human and directly; it is dissipating in all mainlands. The absence of any forestalling antibody demonstrated hazardous to human life. All segments, for example, training, accommodation, transportation, exchanging and so on., and a lot more are not affected by this worldwide pandemic. As of 21st May 2020, out of an aggregate of 4,893,186 affirmed cases, 3,23,256 passings are affirmed in the entire world [2]. A few analysts from the globe have been attempting to foresee Covid-19 cases. Pestilence improvement drifts in South Korea, Italy, and Iran were anticipated utilizing forward expectation and in backward induction [3]. Using bend fitting and repetitive neural system, future Covid-19 positive cases and affirmed cases were distinguished in India [4]. Canada has an aggregate of ten Provinces, and three territories and Covid-19 additionally begin affecting since 30th January 2020 when the main case was seen from Chines inception [5]. The present research study has prime focus on the prediction of Covid-19 cases w.r.t. different parameters such as number of positive cases, number of recouped cases, and decrease cases. The proposed research work will be a beneficial apparatus for overseers, and wellbeing officials to organize the clinical office in the distinctive Canadian Province. The following are the major highlights of the proposed research work:

- Expert modeler forecasting has been proposed for COVID-19 in Canadian Provinces.
- Thirty days of day-ahead estimation for different parameters has been made.
- Analysis of the impact of lockdown, social separation, and effect of change proportion.
- Forecasting and an understanding of conceivable circumstances in the coming days have been reported.
- Predicting of the relationship of deceased patients with recovered and confirmed patients has been made.
- Predicting the decease and confirmed cases in the distinct Provinces.

## Literature Review

**Fig. 1:**
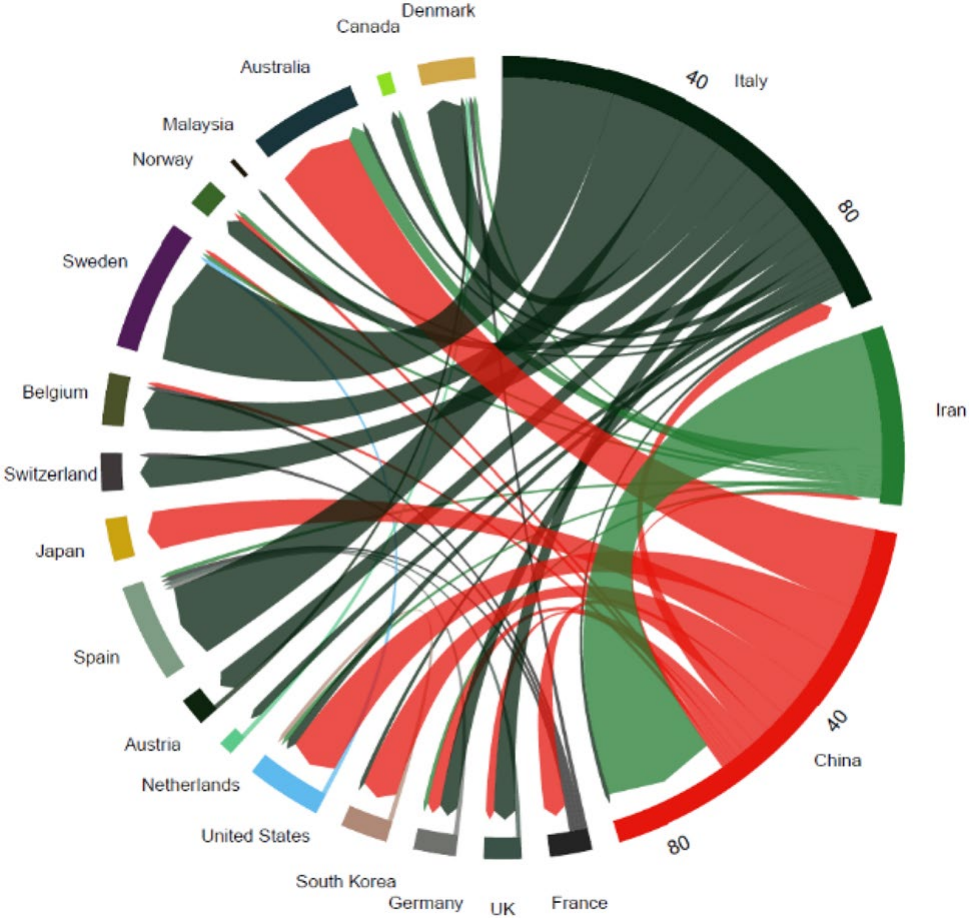
Initial transmissions of Covid-19 between most heavily affected regions

The epidemic comes into picture at wet market (Wuhan city) in China. The first recognized cases have found some symptoms like problem in the respiratory system, sneezing, dry coughing, and fever. But there was no any proof that this virus spread due to this wet market in Wuhan city [6]. Some researchers also said that this virus can also come due to taking the soup of bats, but it is not proven yet. But after sometime this virus spread slowly in more than 34 provinces in China and 25 other countries resulting in 75,199 confirmed cases and 2009 deaths [7]. Then, the World health organization has given a statement that this virus has a tendency to spread from human to human and also called this disease as Coronavirus (Covid-19) [8]. After increasing the number of patients, China started some action plans against fighting this disease like lockdown in the whole country, sanitized all necessary places, and complete quarantine of every citizen. As on 12th May 2020, the world corona meter showing the total number of cases in China is 82,919, from which 4633 have lost their life and 78,171 have recovered from this dangerous virus. Due to the mobility of the people from one country to another this virus has been spread almost all over the world. The total number of cases due to this virus is 4,268,496 from which 287,463 have lost their lives and 1,533,701 have recovered from this virus till 12th May 2020. Now a days, this virus has become a massive threat for all over the world. Because there is no proper drugs and vaccine prepared yet, this virus also drops a massive impact on the whole economy of the world, which is continuously decreasing. Coronavirus has a shell, the particles are round or oval, often pleomorphic, and 50–200 nm in diameter as shown in Fig. 1. S-protein lies on the virus surface, creating a rod-shaped structure [9]. As one of the virus’ most essential antigenic proteins, the S protein gene is the primary target used for typing. Xu et al. also stated that SARS-CoV-2 S-protein supports a strong association with human angiotensin-converting enzyme 2 (ACE2) molecules, which means that S-protein-ACE2 binding pathway poses a major public health risk to human transmission [10]. Awareness of coronavirus physical and chemical characteristics mainly comes from SARS-CoV and MERS-CoV studies. The coronaviruses are heat-sensitive and can be destroyed for 30 min at 56° C. Besides, the virus can be effectively inactivated by ether, 75% ethanol, chlorine disinfectant, peracetic acid, and chloroform, but not by chlorhexidine [11]. Figure 1 depicts the initial transmission of Covid-19 between profoundly affected regions in the world.

Everyday growth rate of affected patients is increasing rapidly. Therefore, keeping this fact in mind, a predictive analysis was performed to estimates the possible count of death of affected patients in Canada. This paper is structured into four sections. Section 1 debated on the introductory theory of Covid-19 with focusing on Canadian impact. Section 2 designed research schema important objectives and relative hypotheses. Section 3 focused on predictive analytical techniques. Section 4 describes the test data under consideration. Section 5 executed experiments with sufficient discussions. Section 6 concluded the major essence of the study.

## Proposed Methodology

We used the SPSS expert model for the forecasting of confirmed and decease cases in the Canadian provinces. Based on specific parameters, it spontaneously recognizes and measures the best-fitting (Autoregressive Integrated Moving Average) ARIMA or exponential smoothing model (ESM) for one or more dependent variable series. We used IBM SPSS statistics 25 tools with the time series module. In this module, a time series is a set of observations obtained by measuring a single variable regularly over time. Based on known previous time-series value in the covid-19 dataset [2], a new model to predict future covid-19 cases are predicted. Below is the standard ARIMA forecasting Eq. (1).

1 $$Y:\; = {\text{ARIMA}}\left( {p,d,q} \right),$$

*p* is the number of autoregressive terms, *d* is the number of no seasonal differences needed for stationarity, and *q* is the number of lagged forecast errors in the prediction equation.

In Eqs. (2), (3), (4)), *y* denotes the *d*th difference of *Y*, which means:

2 $${\text{If }}d = 0: yt = Yt.$$

3 $${\text{If }}d = 1: yt = Yt - Yt - 1.$$

4 $${\text{If }}d = 2: yt = \left( {Yt - Yt - 1} \right) - \left( {Yt - 1 - Yt - 2} \right) = Yt - \, 2Yt - 1 + \, Yt - 2.$$

The general equation of ARIMA shown in Eq. (5), where moving average parameters (θ’s),

5 $$\hat{y}_{t} = \mu \, + \, \phi_{1} y_{t - 1} + \ldots + \phi_{p} y_{t - p} - \theta_{1} e_{t - 1} - \ldots - \theta_{q} e_{t - q} .$$

We made the following Eq. (6) of ARIMA model where *Y* is a dependent variable which is confirmed and *Y*_1_, *Y*_2_, ⋯, *Y*_10_ are Canadian provinces, respectively.

6 $$Y:Y_{1} ,\;Y_{2} ,\;Y_{3} ,\;Y_{4} ,\;Y_{5} ,\;Y_{6} ,\;Y_{7} ,\;Y_{8} ,\;Y_{9} ,\;Y_{10} = {\text{ARIMA}}\left( {p,d,q} \right).$$

The expert model provided the best fit models suggested by ARIMA equation depicted in Table 1.

**Table 1:** ARIMA models for confirmed cases

British Columbia	ARIMA(0, 2, 2)
Grand Princess	ARIMA(0, 1, 8)
Manitoba	ARIMA(0, 2, 1)
New Brunswick	ARIMA(1, 2, 2)
New Fondland and Labrador	ARIMA(1, 2, 0)
Nova Scotia	ARIMA(0, 2, 4)
Ontario	ARIMA(0, 2, 0)
Prince Edward Island	ARIMA(2, 2, 9)
Quebec	ARIMA(2, 2, 0)
Saskatchewan	ARIMA(0, 2, 6)

**Fig. 2:**
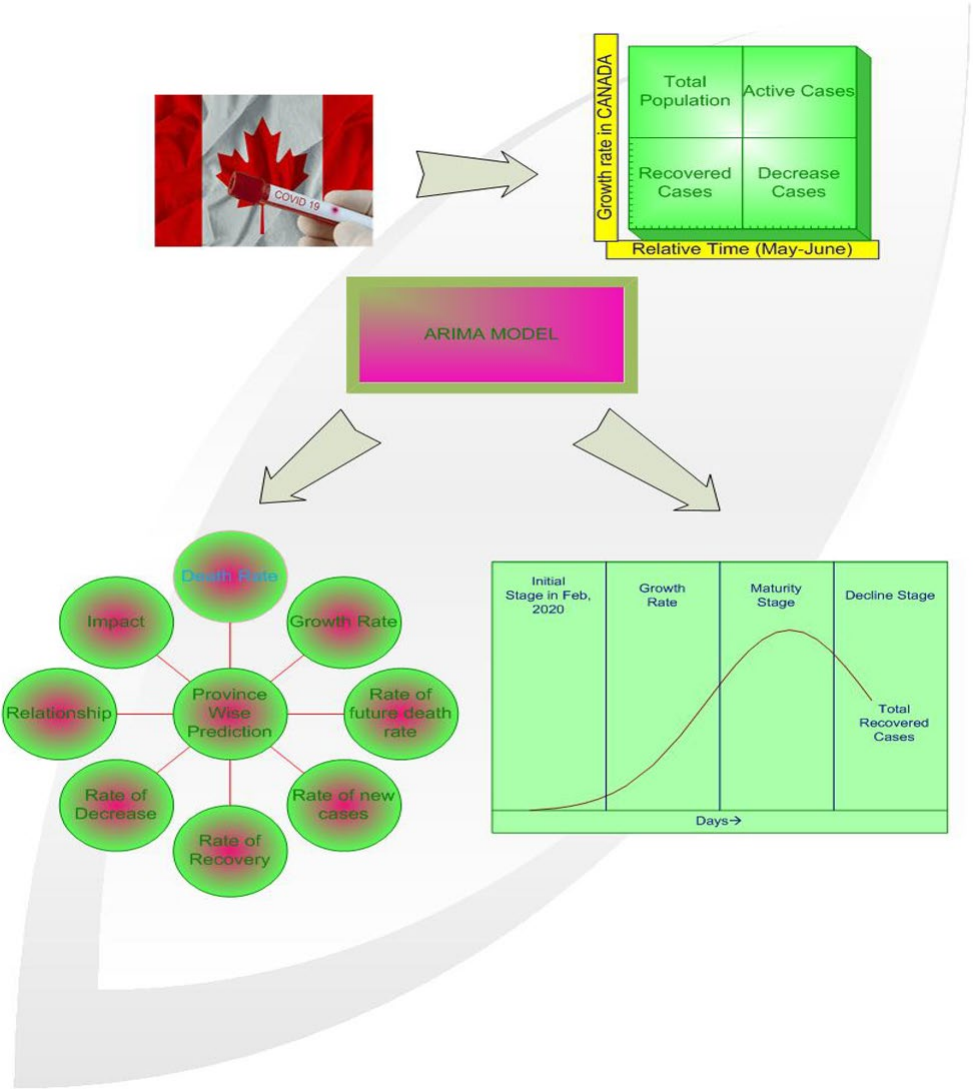
Proposed schematic diagram for Canadian research study

Next Eq. (7) shown the decease prediction where *Y* is decease variable (Fig. 2).

7 $$Y:Y_{1} ,\;Y_{2} ,\;Y_{3} ,\;Y_{4} ,\;Y_{5} ,\;Y_{6} ,\;Y_{7} ,\;Y_{8} ,\;Y_{9} ,\;Y_{10} = {\text{ARIMA}}\left( {p,d,q} \right).$$

## Test Data Under Consideration

**Fig. 3:**
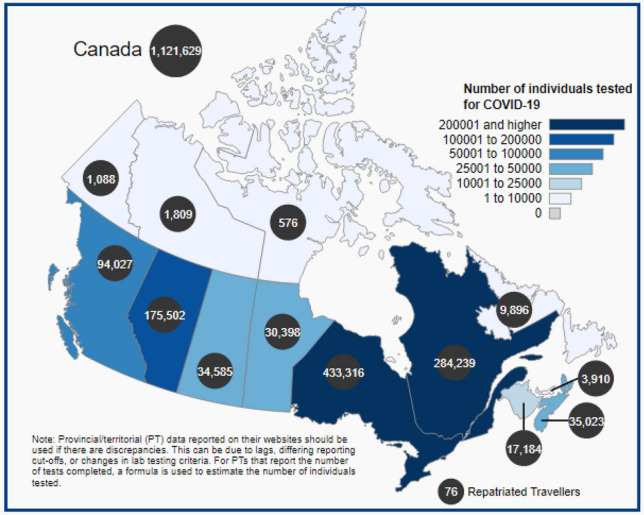
Total number of Individual tested

**Fig. 4:**
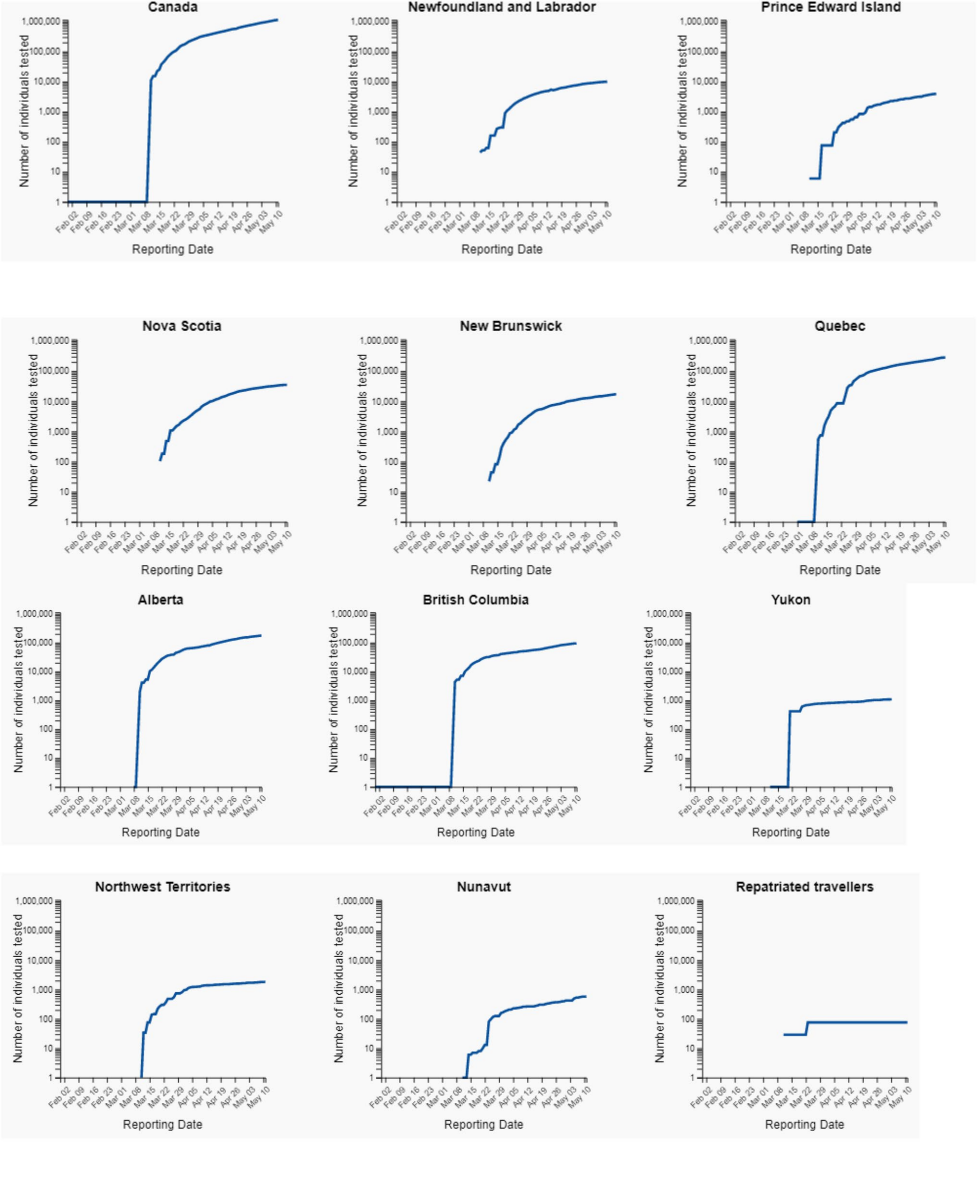
Total number of individual tested up to 11 May 2020 (log scale)

**Fig. 5:**
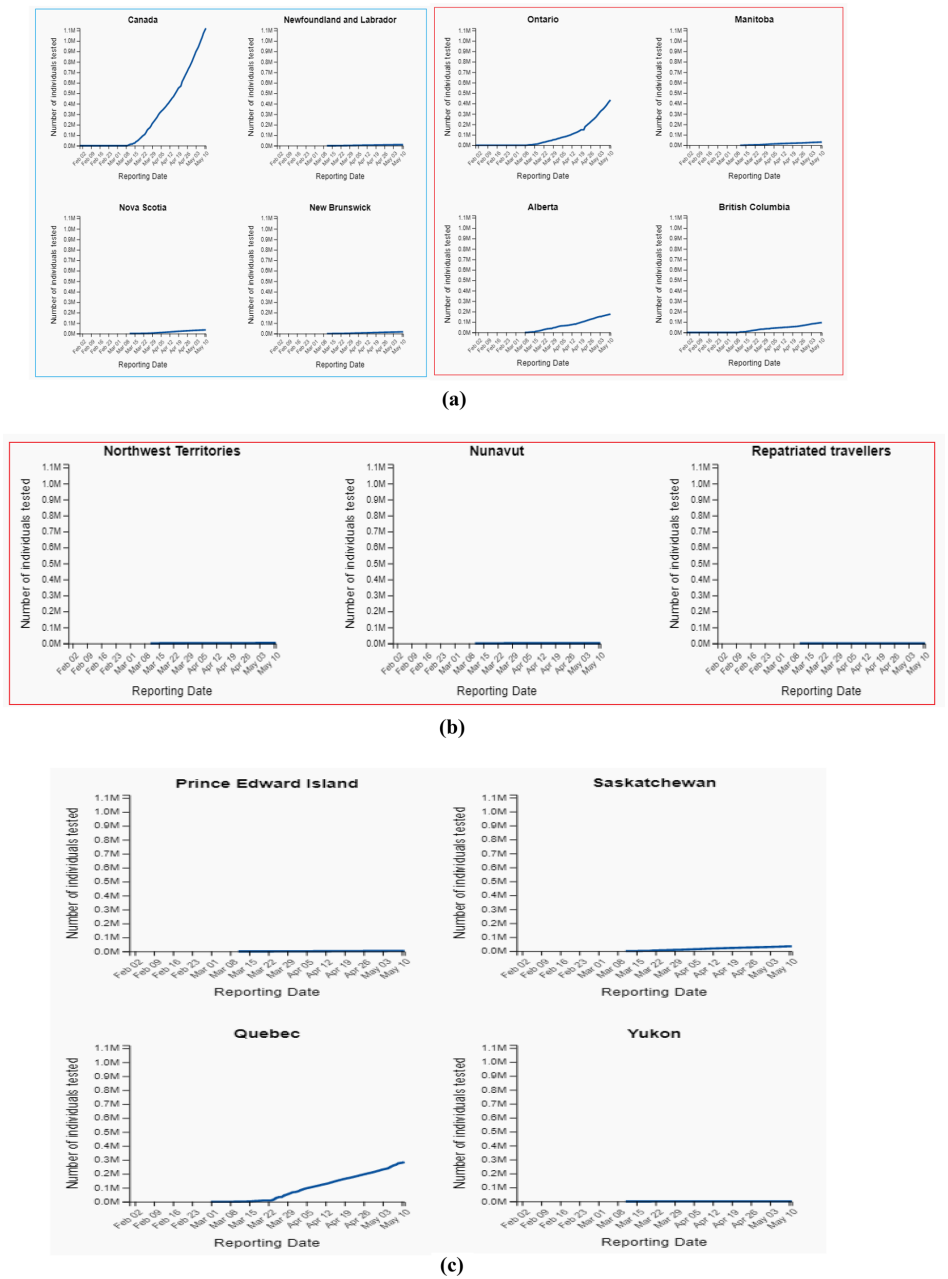
**a** Total number of individual tested upto 11 May, 2020 (normal scale). **b** Total number of individual tested upto 11 May, 2020 (normal scale). **c** Total number of Individual tested upto 11 May, 2020 (normal scale)

The present study used standard and official data set [12] from 12th May to 31 May 2020. The dataset has five major variables named Province, confirmed, recovered, active, and decease. Except for the Province variable, all variables are scale type. The data set is keeping update at the end of each day, having updated records of ten Province and three territories of Canada. The reliability of data samples is calculated by 0.816 using the Cronbach alpha test. Figure 3 depicts the total number of tested individuals up to 11th May, 2020. Table 2 shows the total number of individually tested versus confirmed cases as on 11 May, 2020. Figure 4 depicts the test data for the total number of individuals tested up to 11 May, 2020 (normal scale) (Fig. 5).

**Table 2:** Total number of individual tested versus confirmed cases as on 11 May, 2020

Province, territory or other	Total number of individuals tested	Total number of confirmed cases	Total number of deaths
Canada	11,21,629	69,981	4,993
Newfoundland and Labrador	9896	261	3
Prince Edward Island	3910	27	0
Nova Scotia	35,023	1019	48
New Brunswick	17,184	120	3,013
Quebec	2,84,239	38,469	1,669
Ontario	4,33,316	20,546	7
Manitoba	30,398	289	6
Sakatchewan	34,585	568	117
Alberta	1,75,502	6300	130
British Columbia	94.027	2353	0
Yukon	1088	11	0
Northwest territories	1809	5	0
Nunavut	576	0	0
Repatriated travellers	76	13	0

**Fig. 6:**
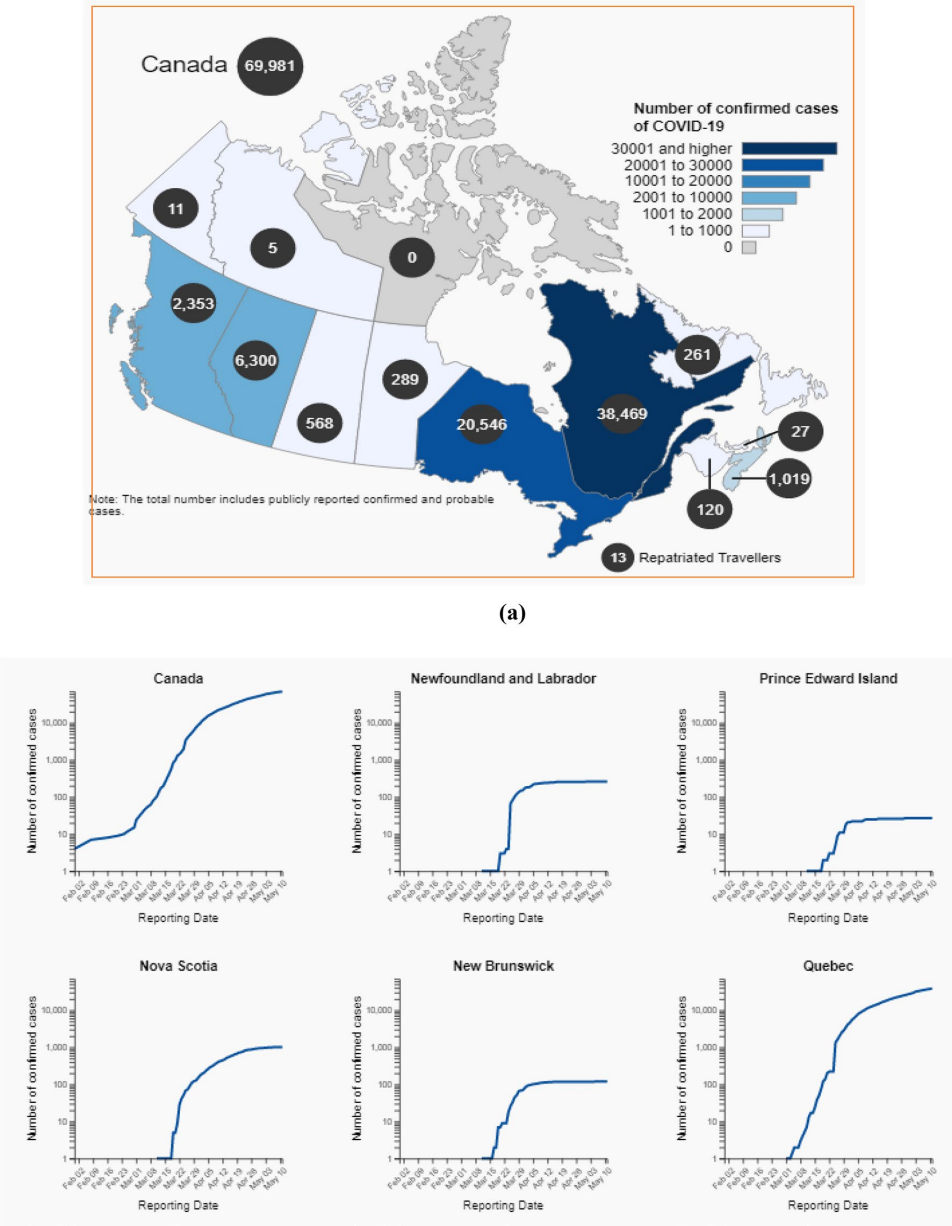

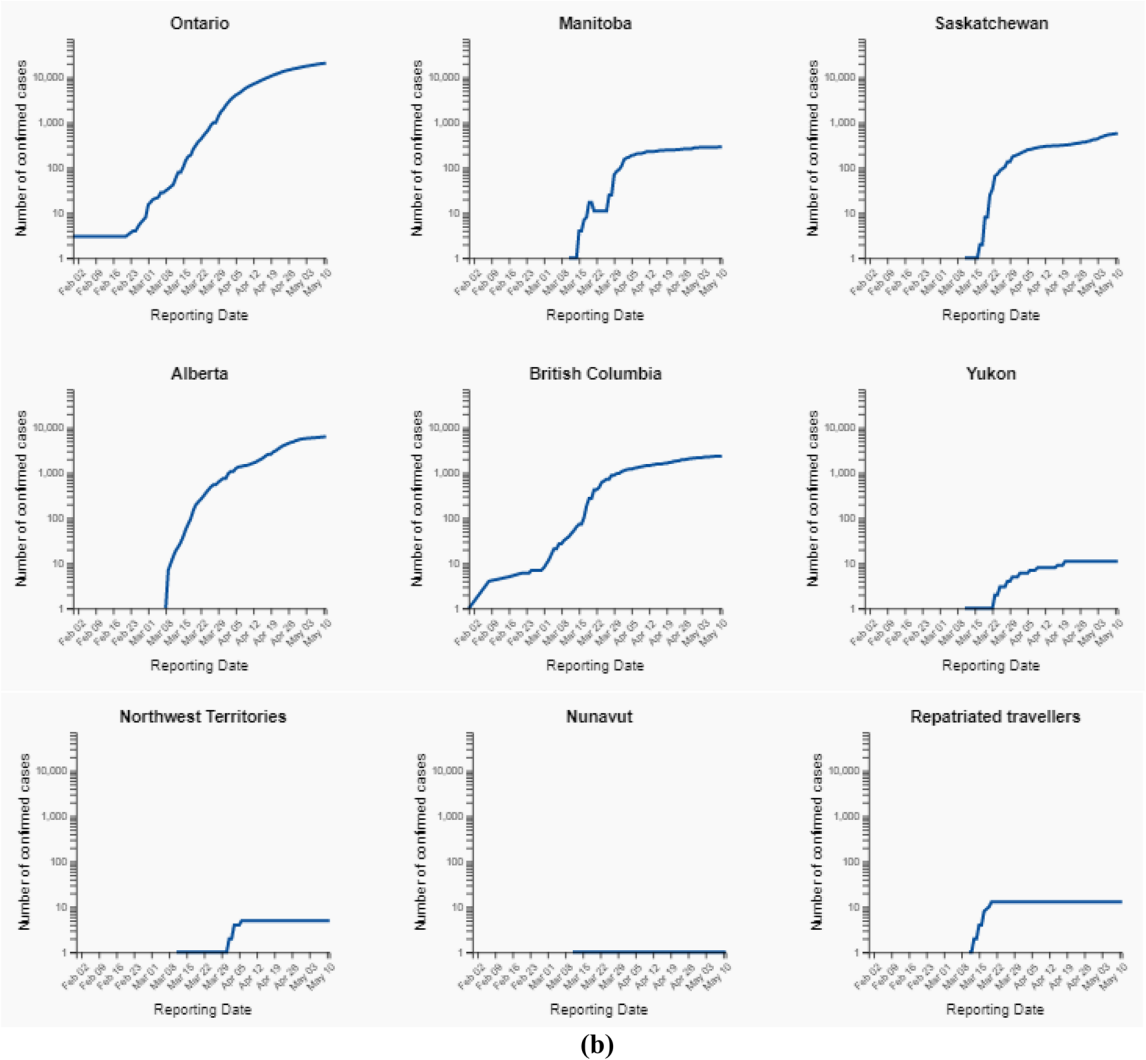
** a** Total number of Covid-19 confirmed cases on 11 May, 2020. **b** Total number of confirmed cases upto 11 May, 2020 (log scale)

Figure 6a, b shows the total number of confirmed cases for covid-19 upto 11th May, 2020 in all the provinces and territories of Canada.

**Fig. 7:**
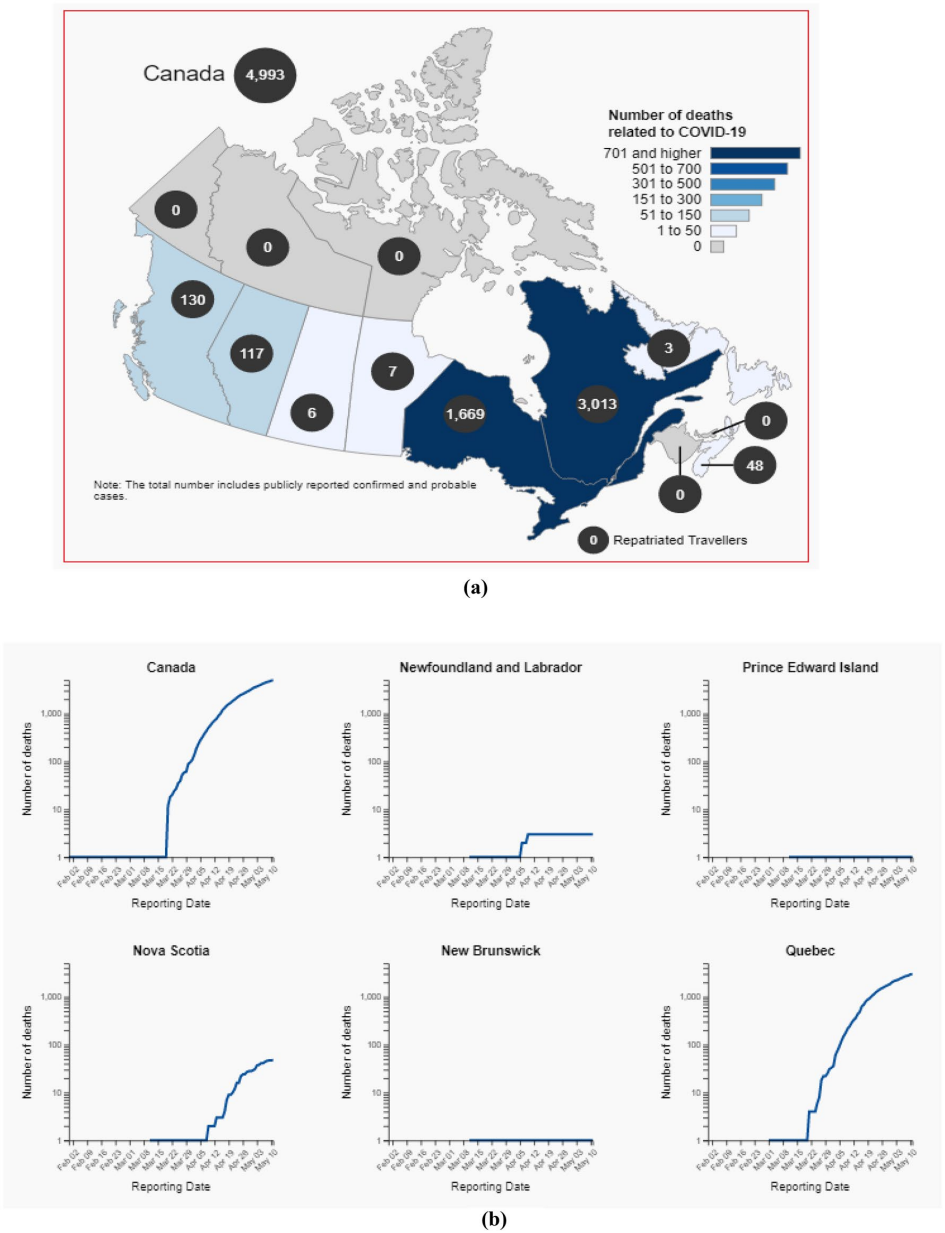

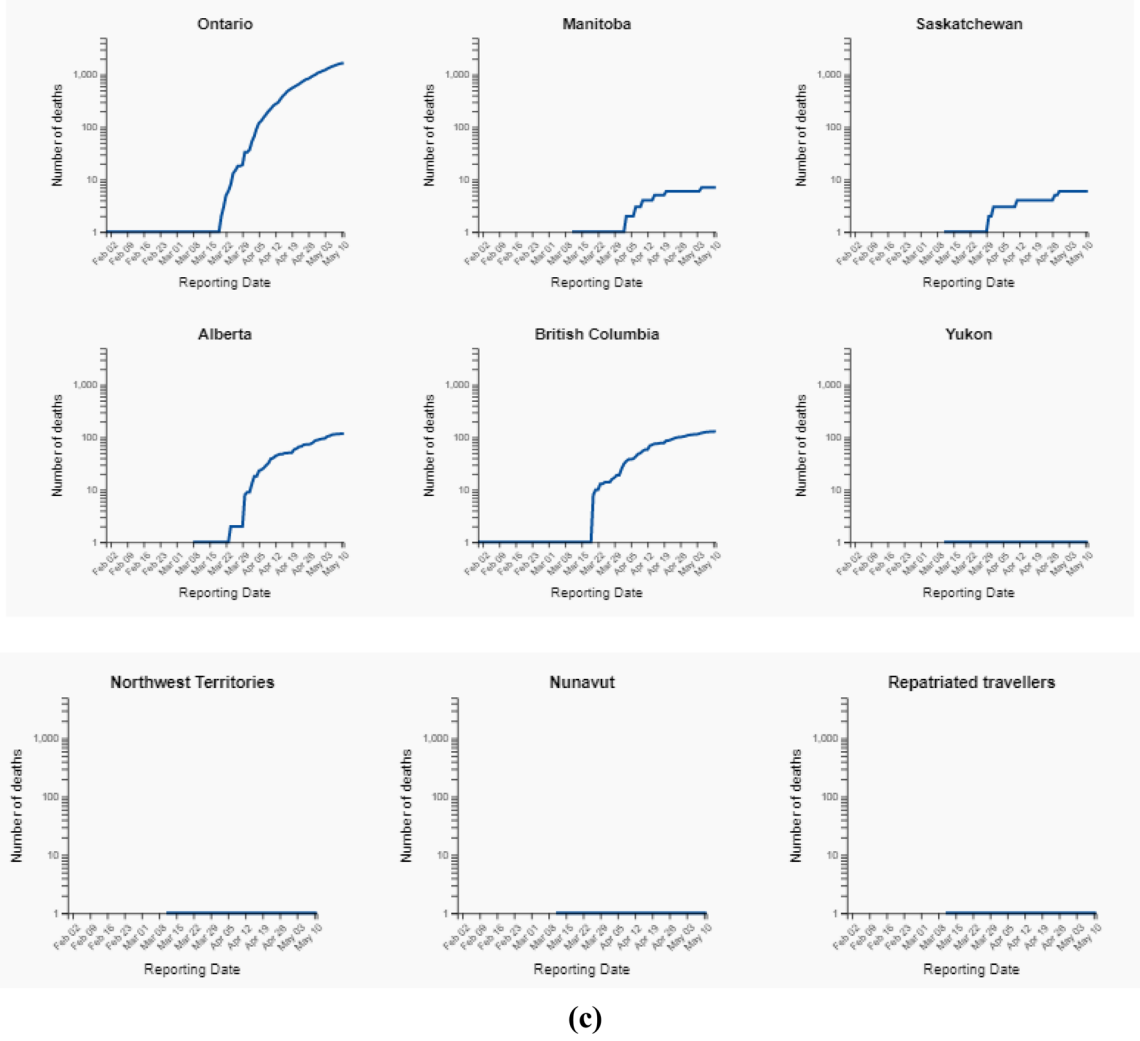
**a** Total number of death cases upto 11 May, 2020. **b** Total number of death cases upto 11 May, 2020. **c** Total number of death cases up to 11 May 2020

Figure 7a–c depicts the total number of decreased cases in all the provinces and territories of Canada.

## Results and Discussion

**Fig. 8:**
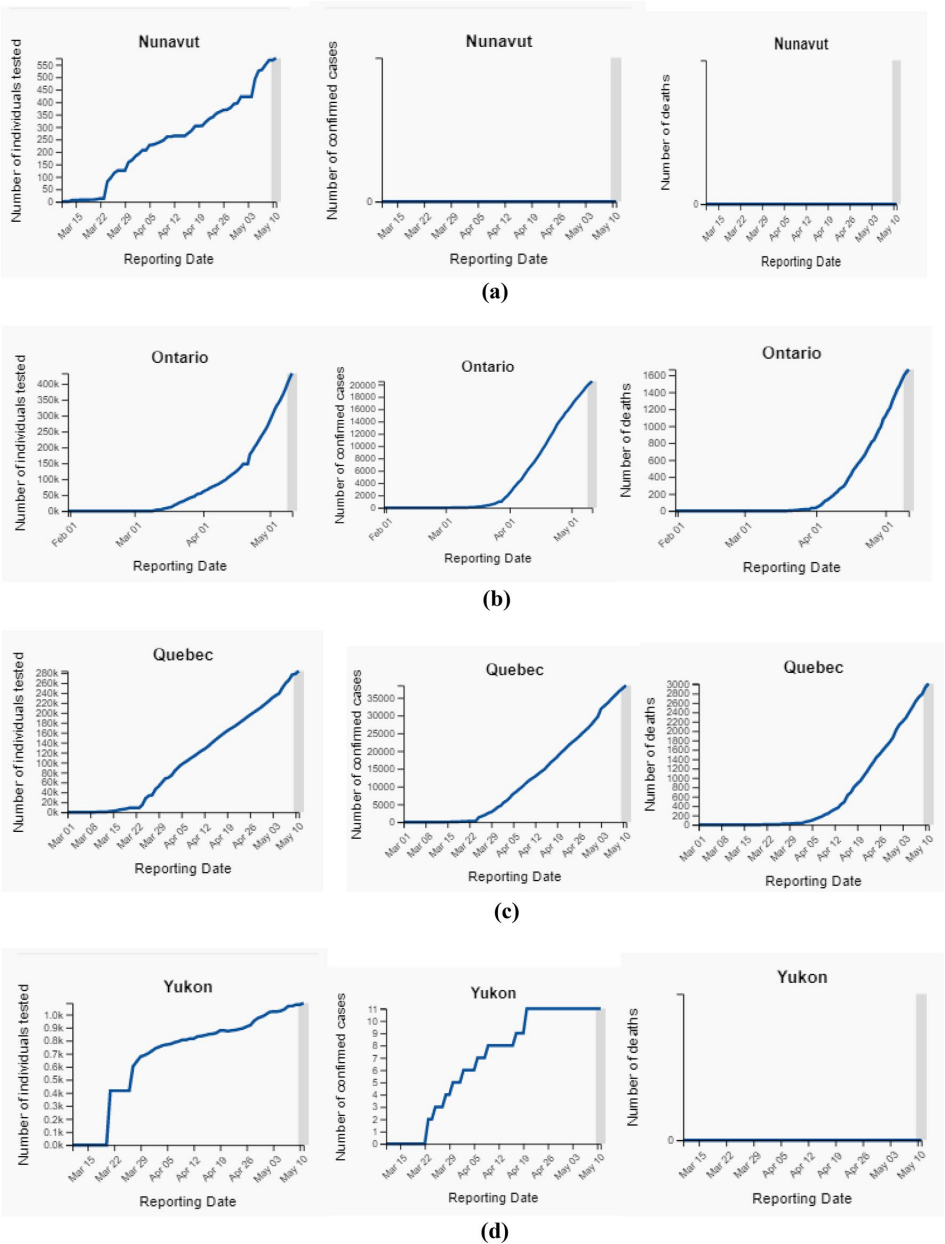
** a** The number of individuals tested, confirmed cases and decreased cases in Nunavut. **b** The number of individual tested, confirmed cases and decreased cases in Ontario. **c** The number of individuals tested, confirmed cases and decreased cases in Quebec. **d** The number of individuals tested, confirmed cases, and decreased cases in Yukon

Figure 8a–d shows the status of the total number of tested, confirmed, and decrease cases in the Canadian Provinces Nunavut, Ontario, Quebec, and Yukon. The maximum number of confirmed cases up to 11th May, 2020 has been recorded are 69,981 and death cases of 4993 with a 7403 deviation value.

The number of individuals tested for COVID-19 in Nunavut was 576 as of May 11, 2020. The number of confirmed cases of COVID-19 in Nunavut was 0 as of May 11, 2020. The number of deaths related to COVID-19 in Nunavut was 0 as of May 11, 2020.

The number of individuals tested for COVID-19 in Ontario was 433,316 as of May 11, 2020. The number of confirmed cases of COVID-19 in Ontario was 20,546 as of May 11, 2020. The number of deaths related to COVID-19 in Ontario was 1669 as of May 11, 2020.

The number of individuals tested for COVID-19 in Quebec was 284,239 as of May 11, 2020. The number of confirmed cases of COVID-19 in Quebec was 38,469 as of May 11, 2020. The number of deaths related to COVID-19 in Quebec was 3013 as of May 11, 2020.

**Fig. 9:**
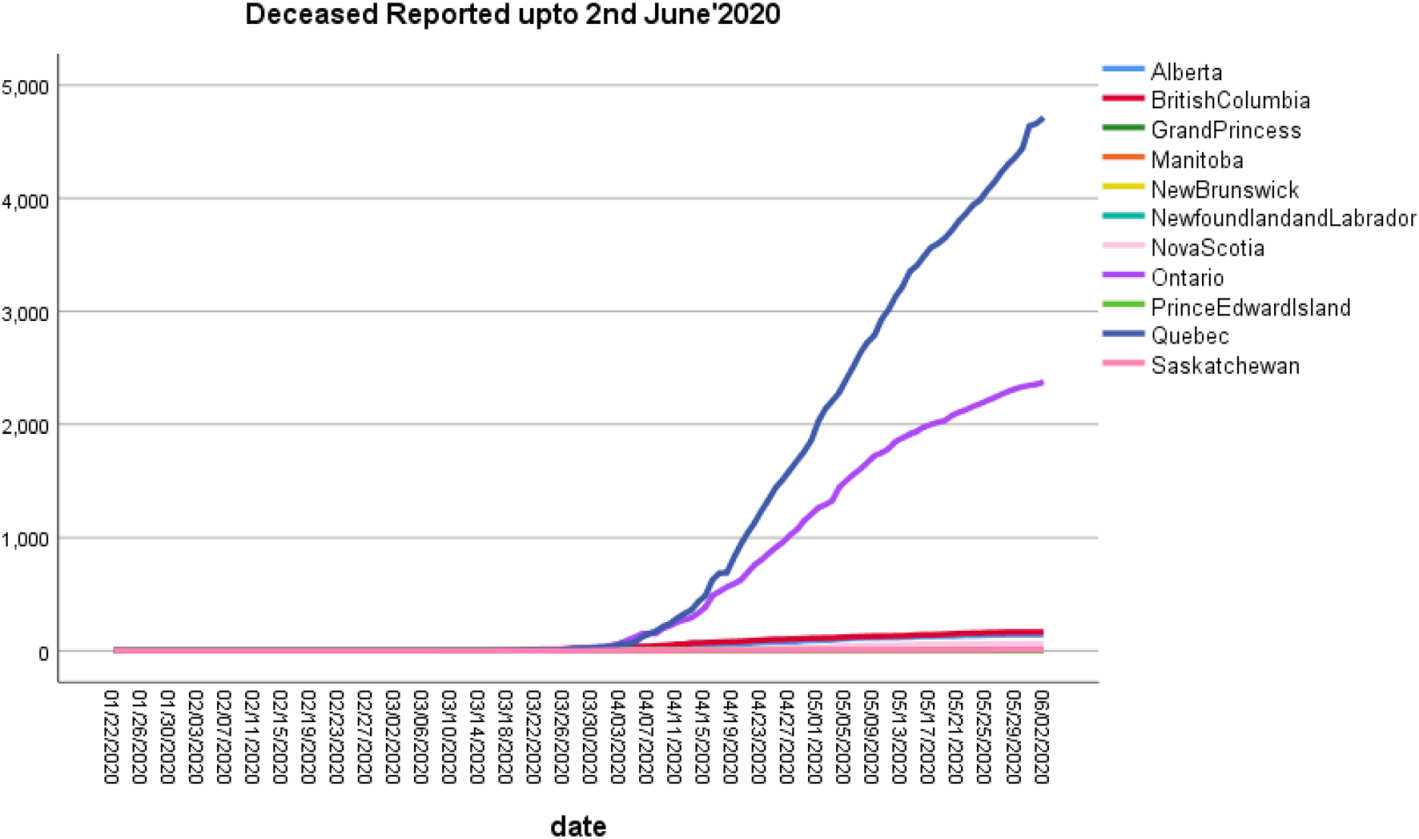
Zone wise total number of decease reported up to 2nd June 2020

The number of individuals tested for COVID-19 in Yukon was 1088 as of May 11, 2020. The number of confirmed cases of COVID-19 in Yukon was 11 as of May 11, 2020. The number of deaths related to COVID-19 in Yukon was 0 as of May 11, 2020. Figure 9 depicts the Zone wise total number of decease reported up to 2nd June 2020. and Fig. 11 shows the decease prospects for the next 27 days up to 30 June 2020.

**Fig. 10:**
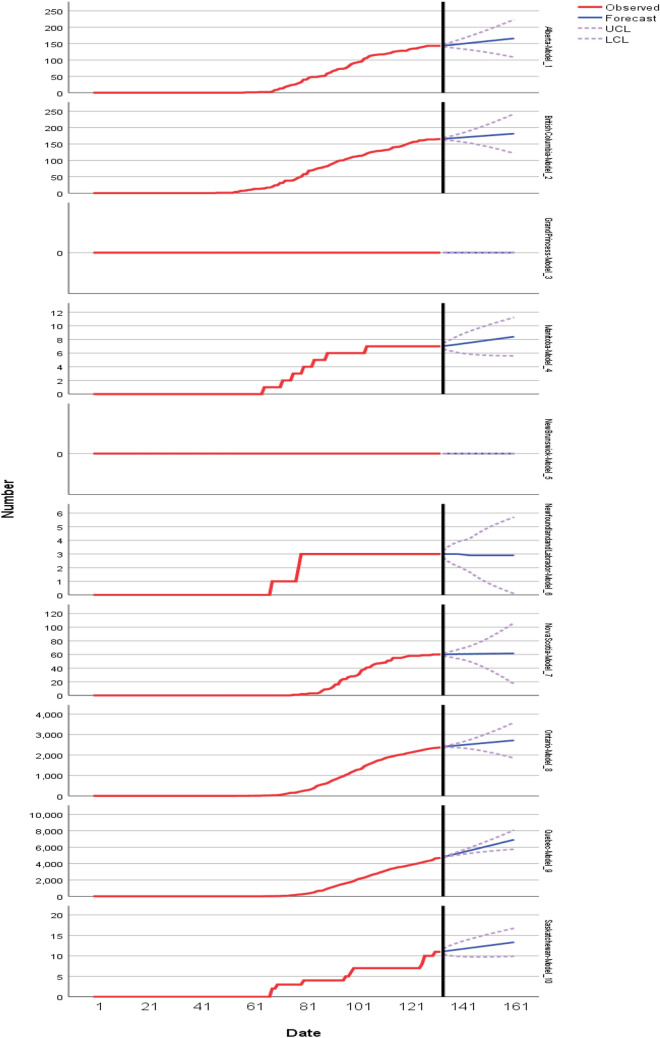
ARIMA Model’s decease prediction for next 27 days up to 30 June 2020

Table 3 shows the model fitting for the decrease. Figure 10 shows the expert model’s for decease prediction for the next 27 days up to 30 June 2020. One the one hand, the blue lines spot forecast and red curve depict the observed cases in the dataset. The expert model proves the forthcoming human deaths may be reported in the Alberta (around 165), British Columbia (around 180), Ontario (around 2700), Quebec (around 6900), Saskatchewan (around 11), and least chances are found in rest of provinces.

**Table 3:** ARIMA model fitting for decease

Fit statistic	Mean	SE	Minimum	Maximum	75	90	95
Stationary *R*-squared	0.368	0.22	− 1.33E−15	0.630	0.557	0.63	0.63
*R*-squared	0.997	0.004	0.990	1.000	1.000	1.000	1.000
RMSE	4.179	8.054	0.000	24.219	4.398	23.086	24.219
MAPE	6.311	1.598	3.785	8.346	7.898	8.346	8.346
MaxAPE	98.980	1.890	95.833	100.000	100.000	100.000	100.000
MAE	2.086	3.976	0.000	11.946	2.321	11.394	11.946
MaxAE	22.272	43.674	0.000	129.902	22.899	124.085	129.902
Normalized BIC	0.521	3.737	− 4.003	6.411	4.134	6.411	6.411

**Fig. 11:**
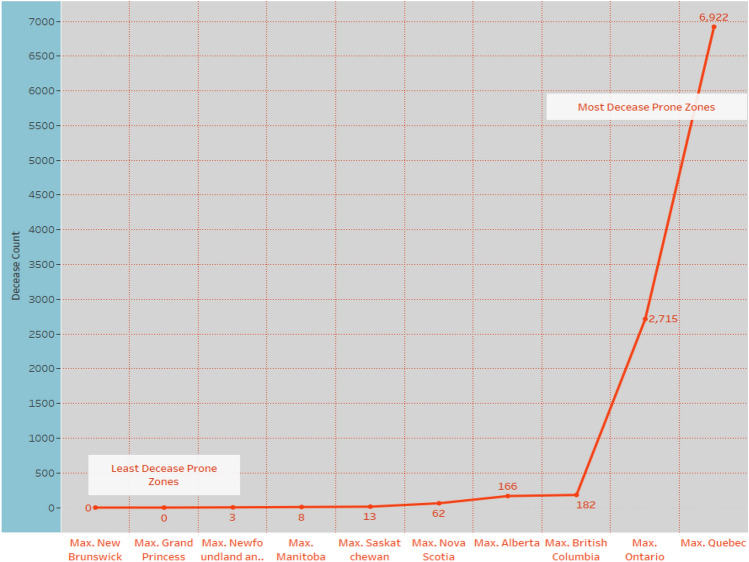
Decease prospects for next 27 days up to 30 June 2020

Figure 11 visualizes the future deceases count in Canadian provinces. We found four provinces (Alberta, British Columbia, Ontario, and Quebec) where future deaths chances are more and entitled most decease prone zones in Canada. Among the four, the highest predicted decease count is 6922 in Quebec. Hence, the republic of Canada needs to more alert and care about the dangerous conditions ahead. According to results, we put three provinces in the least deceases prone zones depicted left side of the graph.

**Fig. 12:**
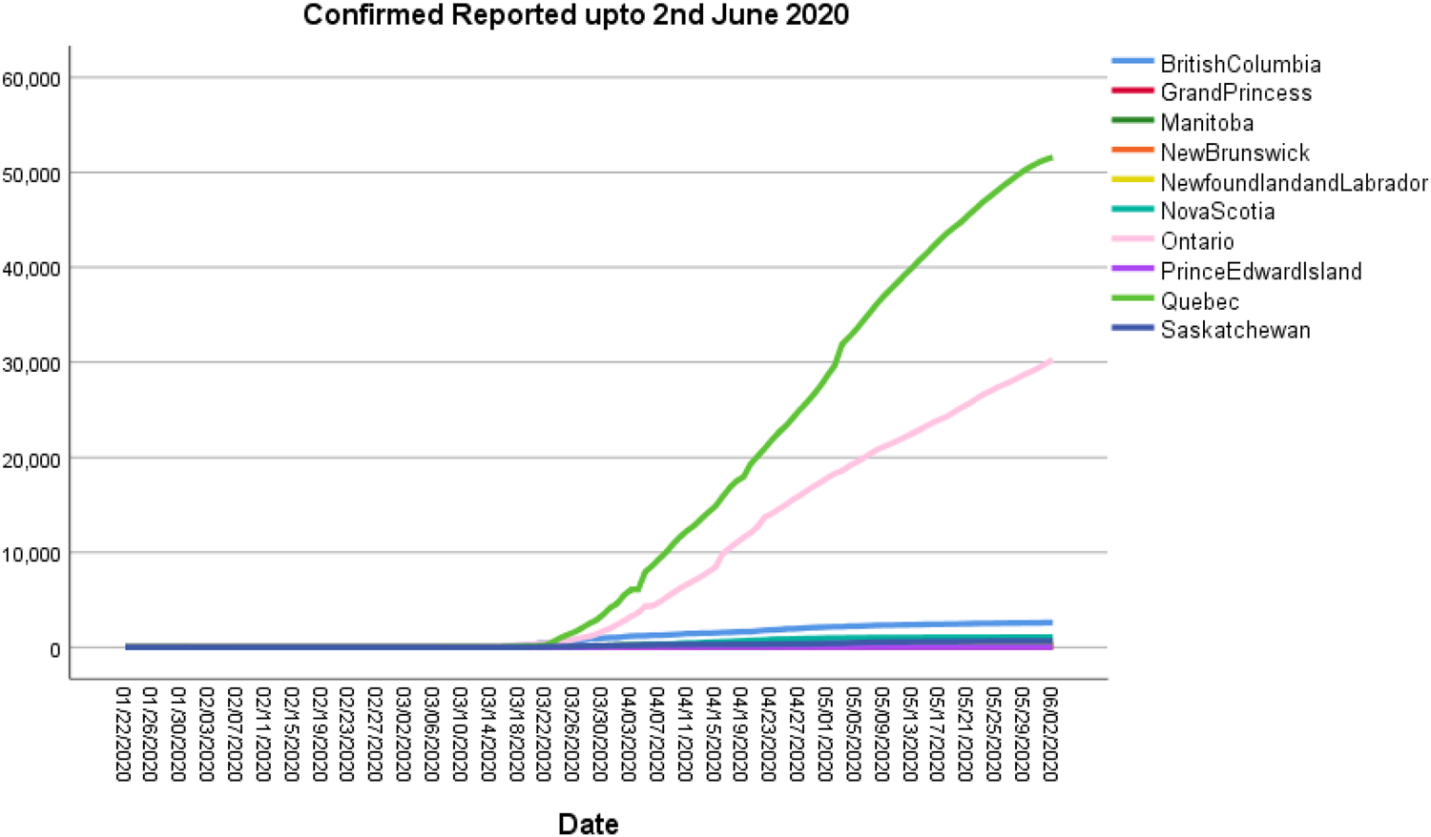
Confirmed individuals up to 02nd June 2020

Figure 12 plots date wise status of confirmed cases reported in distinct provinces of Canada. More than 50,000 humans are infected with covid-19 in Manitoba, and Ontario has around 30,000 cases, which is massive count confirmed cases. Less than 10,000 cases are reported in other provinces. Hence, highly red zone provinces are found in Manitoba and Ontario.

**Fig. 13:**
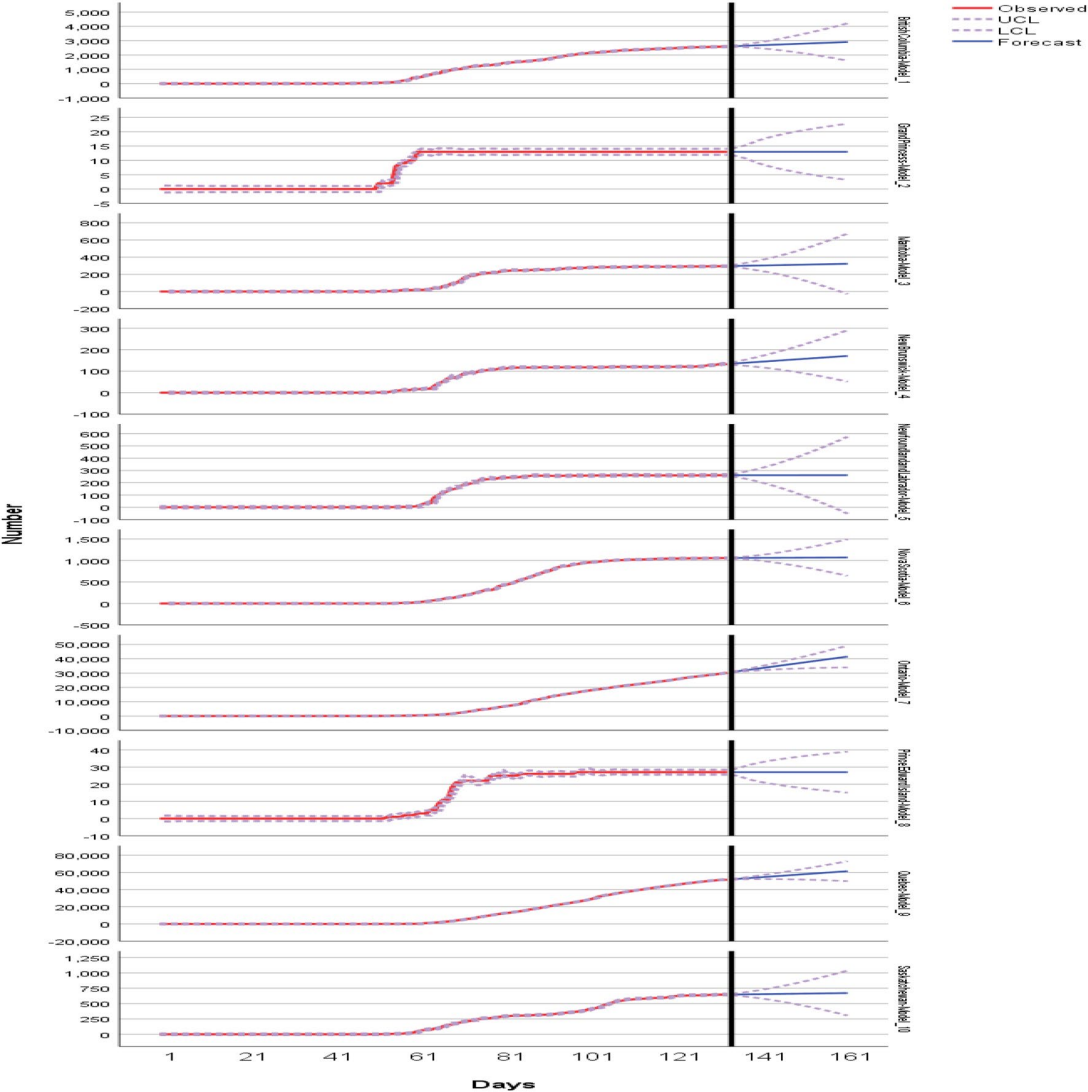
ARIMA Model’s confirmed prediction for next 27 days up to 30 June 2020

**Fig. 14:**
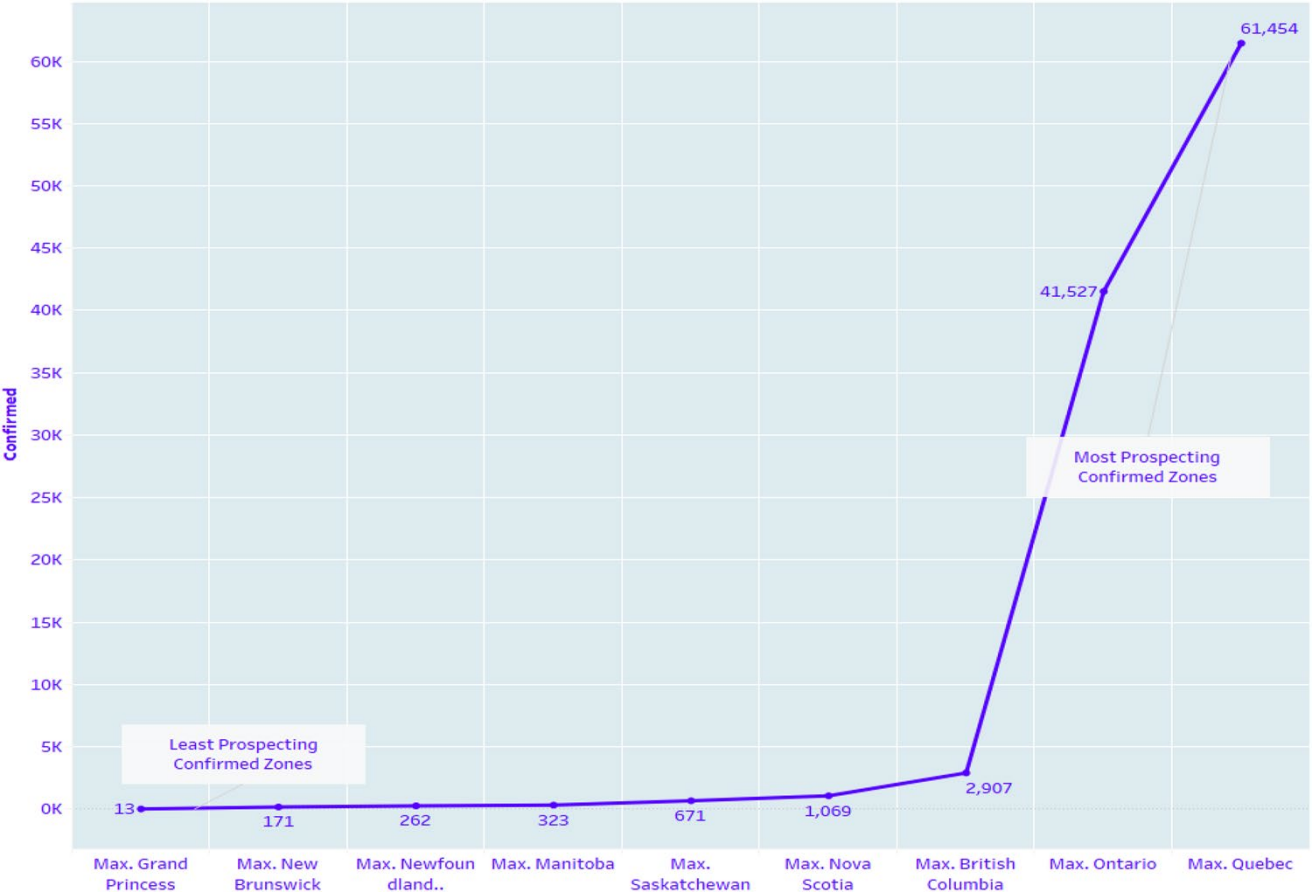
Confirmed prospects for next 27 days up to 30 June 2020

Figure 13 shows confirmed predictions for the next 27 days up to 30 June 2020. Around 3000 confirmed cases may be reported in British Columbia, and Ontario may face around 40,000 confirmed cases. Figure 14 displays the predicted count of the number of confirmed cases in the nine provinces. Four provinces are predicted where huge confirmation is seen like Nova Scotia, British Columbia, Ontario, and Quebec. The least prediction counts are observed in the least prospecting confirmed zones. Table 4 shows the model fitting for confirmed cases in all the provinces of Canada, including territories.

**Table 4:** ARIMA model fitting for confirmed cases

Fit statistic	Mean	SE	Minimum	Maximum	75	90	95
Stationary *R*-squared	0.436	0.129	0.250	0.700	0.490	0.680	0.700
*R*-squared	0.998	0.002	0.993	1.000	1.000	1.000	1.000
RMSE	38.582	71.832	0.533	212.54	48.66	203.724	212.54
MAPE	5.972	1.508	2.808	7.726	7.24	7.717	7.726
MaxAPE	99.540	1.005	97.138	100.000	100.000	100.000	100.000
MAE	17.644	32.157	0.101	95.422	23.901	91.452	95.422
MaxAE	245.591	458.86	4.362	1263.023	315.891	1230.92	1263.02
Normalized BIC	4.047	3.931	− 1.148	10.755	7.206	10.648	10.755

## Conclusion

This paper presented a predictive model to estimate the future human decease infected with Covid-19 for all the provinces of Canada, including three territories. The findings of the study explored the strong relationship between patient decease rate with recovery and active status. On one hand, active cases impacted the decease rate, and on the other hand, patient deceases are effected by the recovered patient. Following the government Covid-19 guidelines, yet to be enhancement are observed in active, recovered, and decease count. Therefore, highly decease prospects are found in the active and recovered cases. Further, present models alerted red zone states to take precautionary measures from the pandemic (Table 5).

**Table 5:** ARIMA models for decease cases

British Columbia	ARIMA (2, 2, 1)
Grand Princess	ARIMA (1, 2, 1)
Manitoba	ARIMA (0, 0, 0)
New Brunswick	ARIMA (0, 1, 4)
New Fondland and Labrador	ARIMA (0, 0, 0)
Nova Scotia	ARIMA (1, 1, 11)
Ontario	ARIMA (1, 2, 10)
Prince Edward Island	ARIMA (0, 2, 5)
Quebec	Brown
Saskatchewan	ARIMA (0, 1, 0)
